# Inadvertent Arterial Cannulation and Norepinephrine Infusion Due to a Misplaced Central Venous Catheter

**DOI:** 10.7759/cureus.17757

**Published:** 2021-09-06

**Authors:** Ali S Al-Shareef, Aida Darweish, Bader Shirah

**Affiliations:** 1 Department of Emergency Medicine, King Abdulaziz Medical City, Jeddah, SAU; 2 Research Office, King Abdullah International Medical Research Center, Jeddah, SAU; 3 College of Applied Medical Sciences, King Saud bin Abdulaziz University for Health Sciences, Jeddah, SAU; 4 College of Medicine, King Saud bin Abdulaziz University for Health Sciences, Jeddah, SAU

**Keywords:** subclavian vein, norepinephrine, central venous cannulation, ischemia, misplacement

## Abstract

Central venous catheter (CVC) insertion is one of the most common procedures done for critically ill patients. The subclavian vein is the particular preferred site. Misplacement of a CVC via the subclavian vein is frequent and can result in life-threatening complications. We aim to report a rare complication of misplaced CVC in the left subclavian vein and norepinephrine infusion that was associated with right upper limb ischemia. Immediate recognition and intervention are key to prevent further complications. The use of ultrasound has proven to reduce such complication, and the hospital implemented the use of ultrasound prior to any CVC placement.

## Introduction

Emergency Department (ED) boarding of critically ill patients is increasing. As a result, more critical care procedures and management are provided in the ED rather than the Intensive Care Unit (ICU) [1]. Central venous catheter (CVC) insertion is one of the most common procedures done for critically ill patients for the purpose of emergency fluid/blood volume restoration, measurement of central venous pressure, administration of toxic drugs, and long-term hyperalimentation [2].

The sites for inserting a CVC are the internal jugular, subclavian, and femoral veins [3]. The most common adverse events associated with a CVC insertion have been extensively addressed in the literature and include infection (5% to 26%), hematoma (2% to 26%), and pneumothorax (up to 30%) [4]. Other complications of CVC placement include hemothorax, chylothorax, extravasation of infusate, unrecognized arterial placement, cardiac tamponade, and mediastinal hemorrhage [4].

Subclavian vein for central access in the particular preferred site due to anatomic location as it has a large diameter, absence of valves, and ability to remain patent and in a relatively constant position [5]. Subclavian catheterization also carries a lower risk of catheter-related infection and thrombosis than femoral or internal jugular vein catheterization [6]. The procedure can be done through two approaches either infraclavicular or supraclavicular. This supraclavicular route to the subclavian vein has some distinct advantages over the infraclavicular approach; such as a well-defined insertion landmark, a shorter distance from skin to the vein, and a larger target area. In addition, the supraclavicular approach less often necessitates interruption of cardiopulmonary resuscitation or tube thoracostomy than the infraclavicular method [5].

Misplacement of the central line via the subclavian vein is relatively common particularly from the left subclavian due to the necessity to traverse corners [7]. Therefore, immediate post insertion X-ray screening is of great importance [7]. Providing such misplacements are recognized early, no problems usually ensue. In most circumstances, a mispositioned CVC should be repositioned or removed as soon as practicable. For some special cases, patients with difficult venous access and a ‘precious’ catheter, an individual risk-benefit analysis should be made about retaining and using the catheter. However, in the literature, optimal management of CVC-related comorbidity is not clear [7]. Immediate removal of the line may be the best option, but consideration must be given to the complications of re-insertion if the patient has an ongoing requirement for central venous access [7]. In this report, we describe a misplaced left subclavian catheter and norepinephrine infusion that led to upper extremity ischemia.

## Case presentation

**Figure 1:**
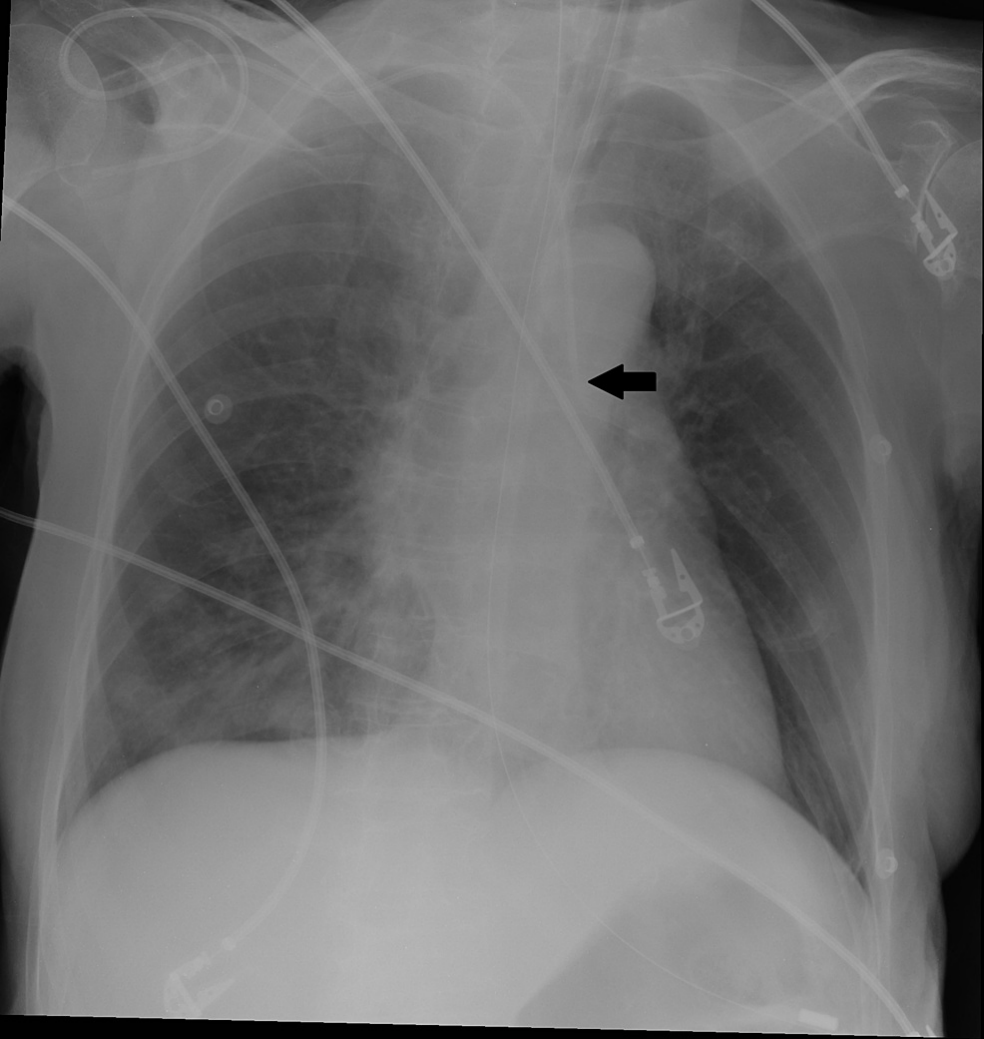
Chest X-ray confirming the placement of the central line

A 76-year-old female presented to the ED with family members via private car. On arrival, she was unresponsive with 3 mm pupils, tachypneic (RR= 30), tachycardic (PR=117), hypotensive (80/40 mmHg), and bedside capillary blood glucose was high. She had a background history of diabetes, hypertension, recurrent strokes, and she was bedridden. The patient was attended by a board-certified emergency physician. She was intubated immediately and connected to a mechanical ventilator. She was resuscitated with two liters of crystalloid fluids for low blood pressure and then vasopressors using norepinephrine as well as insulin infusion as her blood sugar was high. The team was able to insert three peripheral intravenous lines at the following sites: right antecubital, left antecubital, and right hand. As her blood pressure and perfusion were not responding to initial crystalloid and low dose norepinephrine, the attending physician decided to insert a left subclavian catheter where a 7.0-French triple lumen CVC was inserted in the left subclavian vein using infraclavicular approach and anatomical landmarks. The norepinephrine was connected to the CVC, and the dose was increased. Chest X-ray showed that the tip of the central line appears to be in the descending aorta as the line seems to have been inserted into the subclavian artery (Figure 1).

**Figure 2:**
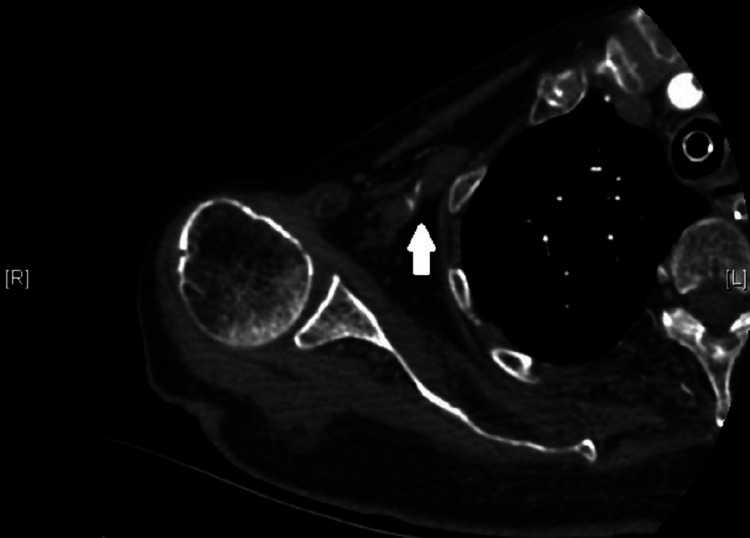
Computed tomography scan showing arterial thrombosis

The norepinephrine infusion was stopped, and the central line was removed with compression. She was transferred from the ED to the medical imaging unit for brain computed tomography (CT) then straight admission to the ICU. During the ICU stay and 8 hours since ED arrival, her right upper extremity was found to be cyanosed in color and pulseless. One day later, CT angiography showed a thrombus with complete occlusion in the right subclavian artery (Figure 2).

The patient’s condition was discussed with a multidisciplinary team including a critical care physician, vascular surgeon, and an interventional radiologist. All had agreed that she is high-risk for surgical intervention, fibrinolysis, and anticoagulation, which was limited given her severe coagulopathy. She remained in the ICU intubated and mechanically ventilated until her demise. 

## Discussion

In this case report, there is a gap evident on how the patient was managed after the subclavian arterial cannulation was identified from the immediate intervention to further investigations required. Despite the discovery of intra-arterial cannulation, unfortunately, the norepinephrine was left infusing for at least another hour, and no further monitoring of the affected limb was carried out. With her poor neurological status, she was not able to verbalize or complain of pain, which is usually the early sign of developing ischemia due to intra-arterial drug administration [8]. The administration should be immediately stopped when an intra-arterial drug injection is suspected [9].

Norepinephrine functions as a peripheral vasoconstrictor (alpha-adrenergic action) and as an inotropic effect on the heart and dilator of coronary arteries (beta-adrenergic action). The initial response to intra-arterial injection of medication is edema followed by congestion of the vessels and swelling of the endothelial linings, damage to the intima, stasis of the blood, and thrombus formation [10]. The intra-arterial injection of drugs would also cause acute, severe extremity ischemia and gangrene [10]. Furthermore, an algorithm was developed for the management of catheter-related cardiothoracic arterial trauma and recommended considering immediate imaging to assess the extent of arterial injury [11]. Despite the patient's situation and critical condition, a CT angiography could have been performed at the same time when the patient was transported to the medical imaging unit from the ED to undergo brain CT. However, the focus was to investigate the presenting condition rather than the iatrogenic complication.

Different methods to confirm thorax CVC position such as chest X-ray, pressure waveform monitoring, ultrasound, fluoroscopy, echocardiography, blood color, and projectile flow have been listed. However, chest x-ray remains the gold standard as it is available in all healthcare facilities [12]. Arterial trauma as a result of misplaced cannulation requires prompt surgical or endovascular management. As a result of this case and upon team review, we recommended providing mandatory teamwork training to all ED staff that incorporates simulation focusing on improving communication and the ability of ED multidisciplinary teams to handle acute situations and adverse events. In addition, we reinforced the use of ISBAR (Identify, Situation, Background, Assessment, and Recommendation) as a standardized and structured communication tool during handovers and when communicating a significant change in patient condition in all clinical departments and nursing units. Furthermore, we developed a hospital-wide clinical practice guideline that defines the central line pre-insertion requirements and post-insertion care that include potential complications. The use of ultrasound has become mandatory for ED physicians prior to CVC insertion. The competency of utilizing ultrasound for CVC insertion has become essential by the hospital to be tested on annual basis. As a result, we have not observed any major complications since the above recommendations were implemented.

## Conclusions

Misplaced CVC to artery can happen. However, immediate recognition and intervention are key to prevent further complications. The use of ultrasound has proven to reduce such complication, and the hospital implemented the use of ultrasound prior to any CVC placement.
